# Genome-wide association study reveals candidate genes relevant to body weight in female turkeys (*Meleagris gallopavo*)

**DOI:** 10.1371/journal.pone.0264838

**Published:** 2022-03-10

**Authors:** Emhimad A. E. Abdalla, Bayode O. Makanjuola, Benjamin J. Wood, Christine F. Baes

**Affiliations:** 1 Centre for Genetic Improvement of Livestock, University of Guelph, Guelph, Ontario, Canada; 2 School of Veterinary Science, University of Queensland, Gatton, Queensland, Australia; 3 Hybrid Turkeys, C-650 Riverbend Drive, Suite C, Kitchener, Canada; 4 Institute of Genetics, Vetsuisse Faculty, University of Bern, Bern, Switzerland; University of Bologna, ITALY

## Abstract

The underlying genetic mechanisms affecting turkey growth traits have not been widely investigated. Genome-wide association studies (GWAS) is a powerful approach to identify candidate regions associated with complex phenotypes and diseases in livestock. In the present study, we performed GWAS to identify regions associated with 18-week body weight in a turkey population. The data included body weight observations for 24,989 female turkeys genotyped based on a 65K SNP panel. The analysis was carried out using a univariate mixed linear model with hatch-week-year and the 2 top principal components fitted as fixed effects and the accumulated polygenic effect of all markers captured by the genomic relationship matrix as random. Thirty-three significant markers were observed on 1, 2, 3, 4, 7 and 12 chromosomes, while 26 showed strong linkage disequilibrium extending up to 410 kb. These significant markers were mapped to 37 genes, of which 13 were novel. Interestingly, many of the investigated genes are known to be involved in growth and body weight. For instance, genes AKR1D1, PARP12, BOC, NCOA1, ADCY3 and CHCHD7 regulate growth, body weight, metabolism, digestion, bile acid biosynthetic and development of muscle cells. In summary, the results of our study revealed novel candidate genomic regions and candidate genes that could be managed within a turkey breeding program and adapted in fine mapping of quantitative trait loci to enhance genetic improvement in this species.

## Introduction

Turkeys are mainly raised for meat and turkey production has increased worldwide in the last few years with the global market for turkey meat increasing to approximately 6 million tonnes per year between 2016 and 2019 [1]. Producing rapidly growing turkeys has been motivating breeders and farmers in response to the global high demand for meat [2, 3]. Thus, improving growth and yield are central to the turkey breeding objectives aimed at increasing production and minimizing costs [4–6]. Studies have shown high positive genetic correlations between turkey body weight at different ages [7, 8]. However, the higher heritability of turkey body weight is between ages 17- and 24-weeks [9].

Genomic selection is a powerful tool in determining selection candidates, but the identification of causative genes underlying genetic variation provides the necessary molecular information for marker-assisted and gene-based selection [7, 8]. Moreover, genetic parameters revealed that selection may not favour all traits of interest [6, 9, 10]. In this context, genome-wide association studies (GWAS) have been widely used to better understand the genetic architecture of complex traits through the identification of quantitative trait loci (QTL) harboring candidate loci.

QTLs affecting body weight have been previously reported in chickens (e.g. [11–13]), pigs [14] and beef [15]. For turkeys, despite several QTLs detected based on QTL mapping [4], no GWAS investigations have been performed to assess body weight. QTL mapping has been useful for detecting QTLs with relatively large effects, however, it does lack power in accurately modeling QTL with small effect, especially for complex traits such as body weight [16]. The current available high-density turkey genomic data opens the door to conduct GWAS, which may boost breeding programs in this species and overcome the unfavorable genetic correlations between traits of interest such as body weight and walking ability [5]. The objective of this study was to identify genetic variants and candidate genes associated with 18-week body weight (BW) in turkeys using GWAS in a turkey population genotyped with a proprietary 65K SNP array.

## Materials and methods

### Ethics statement

This study was carried out in accordance with the principles of the Canadian Council on Animal Care, the Hendrix Genetics Animal Welfare Policy, and the University of Guelph Animal Care Committee. The protocol was approved by the University of Guelph Animal Care Committee (Animal Use Protocol #3782).

### Study population

Phenotypic and genomic data for 24,989 female turkeys from a male line were provided by Hybrid Turkeys, Kitchener, Canada. The birds hatched between 2010 and 2019 and were reared under a standard feeding system with group housing and shared feeders and drinkers. At 18 weeks of age, bodyweight was recorded with an average of 12.91 ± 0.87 kg. Blood samples were collected from each bird to extract DNA using standard industry procedures, and animals were genotyped using a proprietary 65K single nucleotide polymorphism (SNP) array (65,000 SNP; Illumina, Inc.). Markers in non-autosomal regions were removed and missing SNPs were imputed for missing SNPs using Beagle 5.1 [17]. Quality control for the imputed data was performed using PLINK software [18], where SNPs were removed if they had a minor allele frequency lower than 0.05 or significantly deviated from Hardy Weinberg proportions (*P* < 1×10^−8^). The number of markers retained for subsequent analyses was 48,715. More details about this data are presented in Abdalla et al. [5, 19].

### Statistical analyses

Prior to GWAS, principal component (PC) analysis was implemented in PLINK [18]. Using the indep-pairwise option in PLINK, SNP markers were pruned with a window size of 25 markers, a step of 5 markers, and a r^2^ threshold of 0.2. This procedure resulted in 3,891 independent markers which were used to derive the top two PCs. To evaluate the association between SNPs and BW, the following univariate linear mixed was fitted

y=Wα+xβ+u+e,

where **y** is an *n* × 1 vector of phenotypic values for *n* individuals, **W** is an *n* × *c* matrix of covariates (fixed effects) that control the population structure (top 2 PCs) and hatch week-year, **α** is a *c* × 1 vector of the corresponding coefficients, **x** is an n × 1 vector of marker genotypes at the locus being tested, *β* is the effect size of the marker, **u** is an *n* × 1 vector of random polygenic effects with a covariance structure as **u** ~ *N* (**0**, $K\sigma_{g}^{2}$), where **K** is the genetic relationship matrix derived from SNP markers and $\sigma_{g}^{2}$ is the polygenic additive variance, and **e** is an *n* × 1 vector of random residuals with **e** ~ *N* (**0**, $I\sigma_{e}^{2}$), where **I** is an *n* × *n* identity matrix, and $\sigma_{e}^{2}$ is the residual variance component.

Population stratification was assessed using a quantile-quantile (Q–Q) plot in addition to an inflation factor (λ; Yang et al. [20]), which was calculated by dividing the observed median value of the Chi-squared statistic for p-values (obtained from GWAS) by the expected median value of the Chi-squared statistic (approximately 0.456 for 1 df tests). Significant SNPs were determined using a genome-wide false discovery rate (FDR) of 5% [21]. This approach was chosen as it provides a higher power while controlling false discovery rate. The negative logarithm of the P-value for each SNP was displayed in a Manhattan plot and a genome wide line for the 5% FDR was drawn to display significant SNPs. Q–Q and Manhattan plots were generated using qqPlot and Manhattan functions, respectively, available in R [22]. Heritability of BW and phenotypic variance explained by the significant SNPs associated with BW was estimated by fitting a linear model using restricted maximum likelihood implemented in GCTA software [23]. To characterize candidate regions that affect bodyweight, linkage disequilibrium (LD) analysis was performed for the chromosomal regions with multiple clustered significant SNPs using PLINK software [18].

### Assignment of significant SNPs to genes

The Turkey 5.1 assembly [24] was used to assign significant SNPs to genes. In chickens, strong linkage disequilibrium (LD) has been reported to extend up to 10–150 kb [25–27]. In this study, SNPs were assigned to genes if they were located within the genomic sequence of an annotated gene or within 15 kb of the 5’ or 3’ ends of the first and last exons, respectively. This distance is expected to capture proximal regulatory regions and other functional sites that may lie outside but close to the gene such as promoter regions.

## Results and discussion

**Fig 1:**
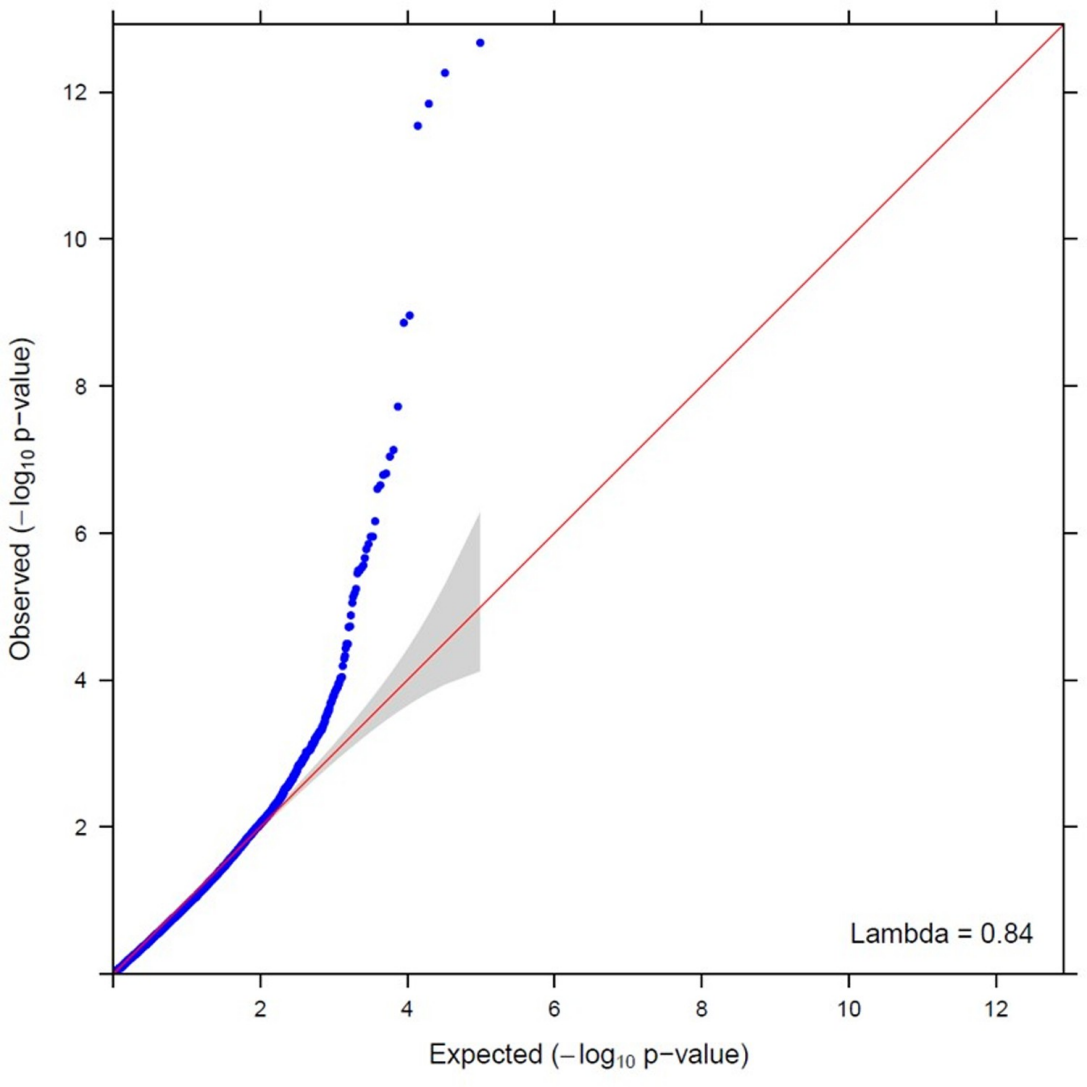
A quantile-quantile plot from the GWAS for 18-week bodyweight in female turkeys using a 65K SNP Illumina array. It shows the late separation between observed and expected values. The genomic inflation factor was 0.84 indicating no population stratification. The shaded area around the quantile-quantile line is the 95% confidence interval (bands).

**Fig 2:**
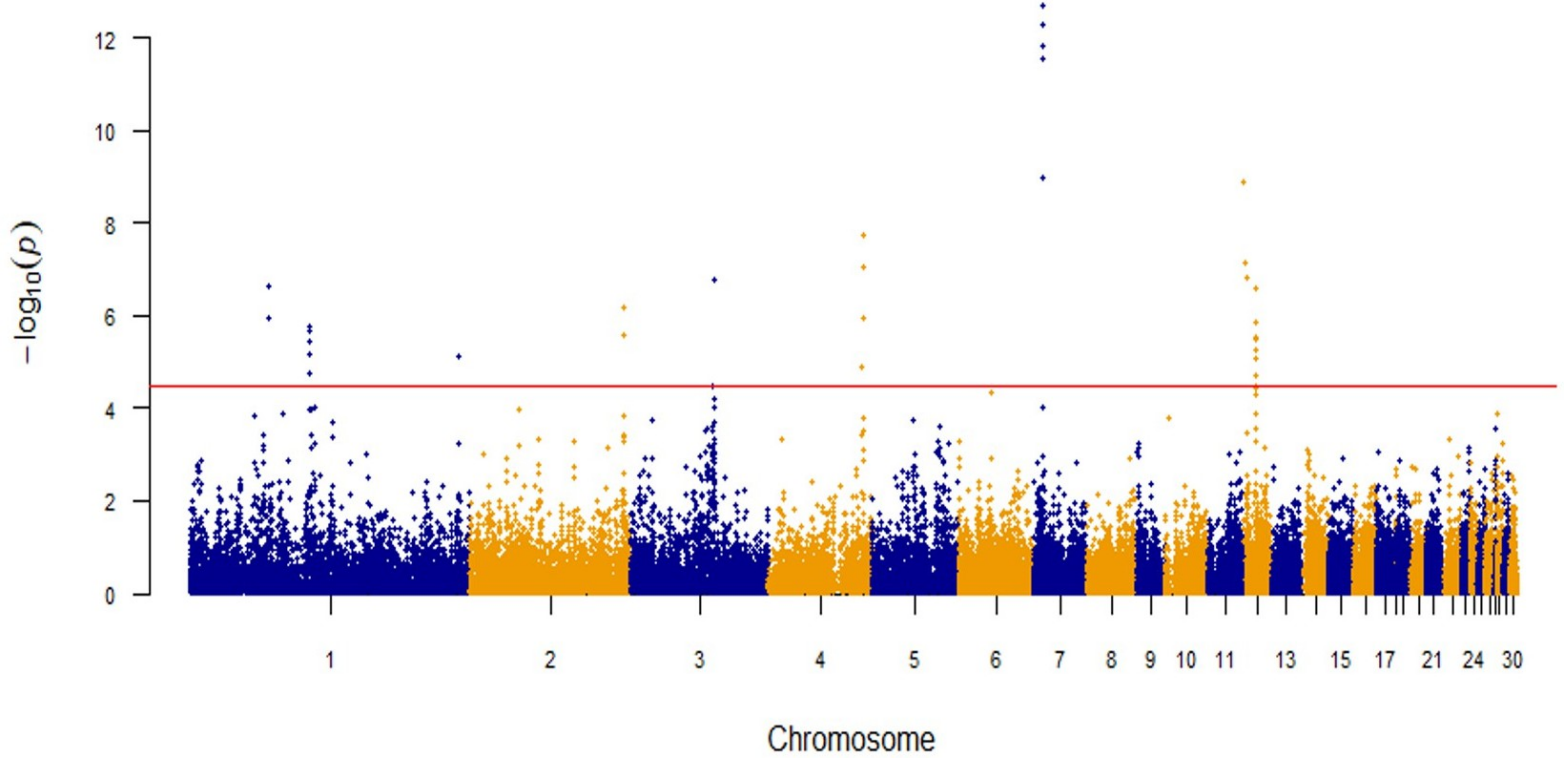
A Manhattan plot from GWAS for 18-weeks bodyweight in female turkeys using a 65K SNP Illumina array. The genomic coordinates of SNPs are displayed along the horizontal axis, the negative logarithm of the association P-value for each SNP is displayed on the vertical axis, and the red line is the significance threshold line at the genome-wise false discovery rate of 5%.

Deviations from the identity line to the left of the Q-Q plot suggest strong association of BW with the SNPs as shown in Fig 1. The genomic inflation factor was 0.84 indicating the absence of population stratification, therefore no adjustment was necessary [28, 29]. Q-Q plots were also obtained after removing the significant SNPs as well as without adjustment for the population stratification. These plots are presented in the supplementary material (S1 and S2 Figs). Based on an FDR of 5%, the Manhattan plot in Fig 2 shows the 33 SNPs that were significantly associated with BW in turkeys and these are shown in Table 1. Among these significant SNPs, 8, 2, 2, 4, 5 and 12 were located on *Meleagris gallopavo* autosomal chromosomes (MGA) 1, 2, 3, 4, 7 and 12, respectively. The minor allele frequency for the significant SNPs ranged between 0.10 and 0.47 and their effect on BW ranged between -0.15kg ± 0.01 and 0.12kg ± 0.03. Heritability of BW was 0.52 ± 0.01 and the phenotypic variance explained by all significant SNPs considered together was equal to 6.9% with a standard error of 0.02. In chickens, the effect of significant SNPs on bodyweight was reported between 3% and 8.1% [30, 31] and our results would appear to be similar to those found in this species.

**Table 1:** SNPs significantly associated with 18-week body weight in turkeys detected by GWAS based on a 65K SNP Illumina panel.

Chr^1^	SNP	Location bp	A1^2^	A2^3^	MAF^4^	Estimated effect	SE^4^	P-value
1	M2013	53847539	G	A	0.47	-0.06	0.02	2.2e-07
1	M2015	53900019	A	G	0.27	0.06	0.02	1.1e-06
1	M2981	81173656	G	A	0.21	-0.08	0.02	3.6e-06
1	M2982	81188549	A	C	0.20	-0.09	0.02	2.2e-06
1	M2983	81222640	A	G	0.20	-0.08	0.02	6.6e-06
1	M2985	81278334	G	A	0.20	-0.08	0.02	1.7e-06
1	M2987	81320624	A	G	0.10	-0.09	0.02	1.8e-05
1	M6708	182307763	G	A	0.26	-0.05	0.01	7.4e-06
2	M10885	104288280	G	A	0.38	-0.06	0.02	6.9e-07
2	M10886	104311936	G	A	0.38	-0.05	0.02	2.8e-06
3	M13167	55687761	G	A	0.21	0.08	0.02	3.2e-05
3	M13195	56416803	A	G	0.36	0.08	0.02	1.6e-07
4	M16706	63330973	C	A	0.41	-0.05	0.01	1.3e-05
4	M16744	64320934	G	A	0.40	-0.06	0.01	1.9e-08
4	M16749	64455195	A	G	0.40	-0.06	0.01	9.0e-08
4	M16750	64475756	G	A	0.37	-0.05	0.01	1.1e-06
7	M23412	7123139	G	A	0.13	-0.14	0.02	1.4e-12
7	M23413	7139162	G	A	0.13	-0.15	0.02	5.6e-13
7	M23414	7146253	C	A	0.13	-0.14	0.02	2.9e-12
7	M23415	7165975	G	A	0.13	-0.15	0.02	2.1e-13
7	M23416	7176912	A	G	0.13	-0.12	0.02	1.1e-09
12	M33976	318853	A	G	0.30	0.08	0.02	1.4e-09
12	M33982	1933794	A	G	0.30	0.08	0.02	7.4e-08
12	M34000	2325482	A	G	0.41	-0.09	0.02	1.5e-07
12	M34622	8352581	G	A	0.42	-0.08	0.02	3.0e-06
12	M34632	8423789	C	A	0.18	0.11	0.03	1.4e-06
12	M34634	8436955	G	A	0.18	0.10	0.03	3.2e-06
12	M34635	8443676	A	G	0.18	0.11	0.03	2.5e-07
12	M34673	8729360	A	G	0.15	0.11	0.03	1.9e-05
12	M34677	8758783	A	C	0.15	0.11	0.03	8.8e-06
12	M34679	8773696	A	G	0.15	0.11	0.03	3.3e-05
12	M34687	8829374	A	C	0.15	0.12	0.03	5.8e-06
12	M34688	8834569	A	G	0.15	0.12	0.03	3.3e-06

^1^Chr = Chromosome

^2^A1 = Major allele

^3^A2 = Minor allele; ^3^MAF = Minor allele frequency

^4^SE = Standard error.

As shown in Table 2, 27 significant SNPs out of the 33 identified in this study were mapped to 37 genes based on the Turkey 5.1 assembly [24] and the 15 kb up- and downstream distances previously described. Some SNPs were mapped to two genes and this is due to the 15 kb up- and downstream distances. From these 37 genes there were 13 novel LOC-named genes, i.e. genes that have yet to be characterised. The remaining six SNPs were neither within nor near any gene (15 kb up- or downstream). The lack of ability to map these SNPs to a gene could be due to the quality of the turkey genome assembly which contains many uncharacterized regions. The failure to connect markers to genes has been previously reported in pigs and was attributed to the low quality of the assembly; and for this reason a wider window of up to 50 kb has been suggested to assign such unlocated SNPs to genes [32]. In the present study, we maintained the 15 kb threshold as we found it a more prudent approach given the smaller relative size of the turkey genome compared to that of other livestock species. Although we also reported the nearest genes to these 6 SNPs in Table 3.

**Table 2:** Genes associated (within 15kbp) with SNPs significantly associated with 18-week bodyweight in female turkeys detected by GWAS based on a 65K SNP Illumina array.

SNP	Chromosome	Position (bp)	Location^1^	Gene name	Entrez
M2013	1	53847539	Within	AKR1D1	100547967
M2015	1	53900019	Within	PARP12	100548121
M2015	1	53900019	3594 D	LOC100548731	100548731
M2981	1	81173656	6068 U	LOC100551192	100551192
M2981	1	81173656	Within	CLDND1	100550575
M2981	1	81173656	13216 D	GPR15	100550729
M2982	1	81188549	8105 U	CLDND1	100550575
M2982	1	81188549	Within	GPR15	100550729
M2982	1	81188549	13123 D	LOC100550884	100550884
M2983	1	81222640	995 U	ILDR1	100551346
M2983	1	81222640	Within	CFAP44	100538354
M2985	1	81278334	Within	BOC	100538508
M6708	1	182307763	Within	UVRAG	100544950
M10885	2	104288280	7357 U	ADCY3	100538467
M10885	2	104288280	310 U	CENPO	100538621
M10886	2	104311936	794 D	NCOA1	100538778
M13167	3	55687761	Within	LOC104910342	104910342
M13195	3	56416803	1232 U	CHCHD7	100538622
M13195	3	56416803	693 U	SDR16C5	100538468
M16706	4	63330973	14095 U	LOC100544079	100544079
M16706	4	63330973	10800 U	LOC116216609	116216609
M16706	4	63330973	78 U	LOC109367489	109367489
M16706	4	63330973	5350 D	LOC100544391	100544391
M16744	4	64320934	5020 U	LOC104909285	104909285
M16744	4	64320934	10844 D	LOC100544235	100544235
M16749	4	64455195	Within	LOC104910910	104910910
M16750	4	64475756	12094 D	SPR	100542371
M23412	7	7123139	11509 U	PMS1	100543933
M23412	7	7123139	8842 D	TRNAL-CAG	109368711
M23413	7	7139162	7099 U	TRNAL-CAG	109368711
M23413	7	7139162	12568 D	MSTN	100303659
M23414	7	7146253	14190 U	TRNAL-CAG	109368711
M23414	7	7146253	5477 D	MSTN	100303659
M23414	7	7146253	10964 D	C7H2orf88	104911634
M23415	7	7165975	8641 U	MSTN	100303659
M23415	7	7165975	Within	C2orf88	104911634
M23416	7	7176912	Within	C2orf88	104911634
M23416	7	7176912	8571 D	HIBCH	100544087
M33976	12	318853	Within	RBPMS2	100539257
M33982	12	1933794	7167 D	HMG20A	100543882
M34000	12	2325482	Within	LOC100549019	100549019
M34622	12	8352581	Within	AP4E1	100539719
M34622	12	8352581	3086 D	TNFAIP8L3	100539875
M34632	12	8423789	Within	LOC100549427	100549427
M34634	12	8436955	2302 U	LOC100549427	100549427
M34634	12	8436955	14598 D	GLDN	100540031
M34635	12	8443676	9023 U	LOC100549427	100549427
M34635	12	8443676	7877 D	GLDN	100540031

^1^Within: the SNP is located within the gene; U: the SNP is located upstream of the gene; D: The SNP is located downstream of the gene.

**Table 3:** Genes associated (more than 15K bp) with SNPs significantly associated with 18-weeks bodyweight in female turkeys detected by GWAS based on a 65K SNP Illumina panel.

SNP	Chromosome	Position (bp)	Location^1^	Gene name	Entrez
M2987	1	81320624	034377 U	BOC	100538508
M34673	12	8729360	106854 U	LOC116217089	116217089
M34677	12	8758783	136277 U	LOC116217089	116217089
M34679	12	8773696	151190 U	LOC116217089	116217089
M34687	12	8829374	179344 D	SEMA6D	100549580
M34688	12	8834569	174149 D	SEMA6D	100549580

^1^Within: the SNP is located within the gene; U: the SNP is located upstream of the gene; D: the SNP is located downstream of the gene.

**Fig 3:**
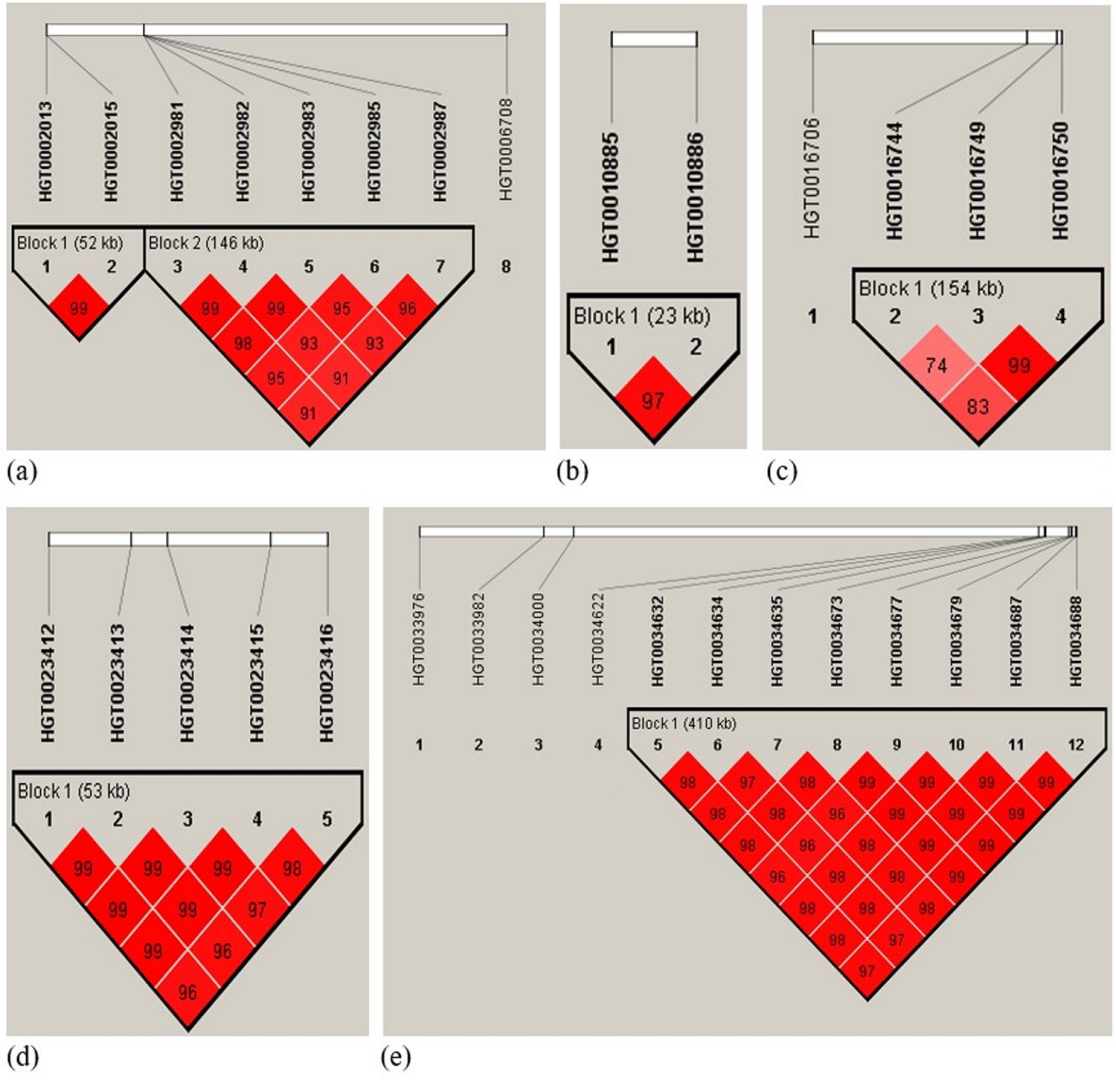
Linkage disequilibrium plots for significant SNPs associated with 18-week bodyweight in female turkeys on Meleagris gallopavo autosomal chromosome (MGA) 1 (a), MGA 2 (b), MGA 4 (c), MGA 7 (d) and MGA 12 (e).

The 8 SNPs significantly associated with bodyweight on MGA 1 were located between 53.8 Mb and 182.3 Mb on the turkey genome shown in Table 1 and found to be distributed into two LD blocks that is depicted in Fig 3A. SNPs M2013, which is the leading SNP, and M2015 had a strong LD constructing a 52 kb long LD block (Block 1; Fig 3A). These two SNPs are located within *AKR1D1* and *PARP12* genes, respectively (Table 2). A third gene, which is the novel gene LOC100548731, is near M2015 and located 3,594 bp downstream of it. The *AKR1D1* gene is a key gene that plays a critical role in the synthesis of bile acid and the metabolism of steroid hormones (e.g. [33, 34]), and studies have shown that dietary supplementation of bile acids can affect the activity of intestinal and lipoprotein lipases leading to improvement of broiler chicken growth [35, 36]. *PARP12* gene, on the other hand, is a polymerase family member and found to be involved in regulating fatty acid metabolism [37] as well as body weight gain and insulin resistance in rats [38].

The second LD block (146 kb) on MGA 1 includes 5 SNPs, once again shown in Fig 3A where the leading SNP was M2985 (Table 1). Except for M2987, the SNPs in this block were both located within genes and near (up- or downstream) other genes within the 15 kb regulatory distance considered in this study. The genes are *LOC100551192*, *CLDND1*, *GPR15*, *LOC100550884*, *ILDR1*, *CFAP44* and *BOC*. The latter was also the nearest gene to M2987 (34 kb upstream; Table 3). The protein encoded by *BOC* mediates cell-cell interactions between muscle precursor cells and promotes myogenic differentiation [39]. Such protein has been reported to be associated with bodyweight gain and obesity in mice [40]. The expression of *CLDND1* gene alters the metabolism functions in the liver leading to the progression of liver diseases [41] and in a recently published study, Zhu et al. [42] indicated that the *CLDND1* gene is associated with energy production and fat metabolism in laying ducks. Moreover, according to Yi et al. (2016), the deficiency of fat metabolism in the liver increases ammonia levels and subsequently growth performance [43] and body fat distribution in broilers [44].

Two significant SNPs on MGA 2 affect bodyweight (Fig 3B); both had a strong LD and were located within a haplotype block span of 23 kb which covers *ADCY3*, *CENPO* and *NCOA1* genes (Table 2). Interestingly, studies have shown that mutation and loss of function in *ADCY3* induces bodyweight gain in humans [45–47]. One LD block (154 kb) for three significant SNPs associated with bodyweight was detected on a candidate region on MGA 4 (Fig 3C) covering the gene *SPR* in addition to three novel genes (Table 2). All significant SNPs on MGA 7 showed a strong LD located with one block, which extends to 53 kb (Fig 3D). Based on the Turkey 5.1 assembly [24], five genes: PMS1, *TRNAL-CAG*, *MSTN*, *C2orf88* and *HIBCH*, were located within this LD block (Table 2). It is noteworthy to mention that the *MSTN* gene encodes a secreted ligand of the transforming growth factor-beta superfamily of proteins and regulators of muscle growth in chickens [48].

Fifteen significant SNPs associated with bodyweight were found on MGA 12, which was the highest number of significant SNPs on a single chromosome in this study (Tables 2 and 3). Five out of these 15 SNPs were not located near any gene within the 15 kb distance (Table 3). The LD analysis indicated that 8 SNPs had a strong LD (r^2^ ≥ 0.97) located within a single 410 kb long LD block. Whereas the first 3 SNPs in this block cover two genes (*GLDN* and *LOC100549427*) within the 15 kb distance (Table 2), the last 5 SNPs were positioned near SEMA6D and LOC116217089 genes but were beyond the 15 kb distance (Table 3). The *HMG20A* gene plays an important role in obesity in human [49, 50] and mice [51]. The *TNFAIP8L3* gene was reported to affect growth and backfat thickness in pigs [52].

Finally, the distance between the two significant SNPs on MGA 3 was more than 729 kb. The first SNP, M13167, was within the novel gene LOC104910342, and the second SNP, M13195, was upstream of *SDR16C5* (693 bp) and *CHCHD7* (1,232 bp) genes. Nishimura et al. [53] indicated that *CHCHD7* was significantly associated with carcass weight in Japanese black cattle, and recently Edea et al. [54] reported that *SDR16C5* gene is associated with weaning weight, yearling weight and bodyweight gain in Korean cattle breeds.

## Conclusions

In this study, we performed a GWA study for 18-weeks bodyweight in female turkeys using a 65K SNP array. The results revealed that 33 SNPs were significantly associated with this trait based on a 5% FDR. The linkage disequilibrium analysis showed that most of these genes are grouped into blocks that extend up to 410 kb. The significant SNPs were mapped to 37 genes, of which 13 were novel. Most of the genes detected are involved in functions related to bodyweight and growth, which has been supported by gene network analyses. The functions of the significant genes included regulation of growth, metabolism, digestion, bile acid biosynthetic and development of muscle cells. These findings could contribute to a better understanding of the genetic architecture of body weight gain in turkeys. However, further examination is required to prove the novel genes discovered in this study as putative genes for body weight in female turkeys.

## Supporting information

S1 Fig A quantile–quantile plot from GWAS for 18-week body weight in turkeys using a 65K SNP Illumina panel for all SNPs (dark blue) and after excluding SNPs significantly (false discovery rate of 5%) associated with the trait (black). The sharp deviation above an expected -log10 p-value of approximately 3 is due to a strong association of 18-week body weight in turkeys with significant SNPs. Exclusion of significantly associated SNPs may leave a residual upward deviation leading to identify more associated SNPs with the trait, which was not the case in this study.
(TIF)

S2 Fig A quantile–quantile plot from GWAS for 18-week body weight in turkeys using a 65K SNP Illumina panel with adjustment for population stratification using the two top principal components (light blue) and without adjustment (dark blue). The adjustment for population stratification did not change the findings of this GWAS study. The population used in this study is a pure turkey line and confounding due to population subgroups is unlikely to be observed.
(TIF)
