# Supplementary figures and images for: Genome-wide association study reveals candidate genes relevant to body weight in female turkeys (Meleagris gallopavo)
Source: PLoS One. 2022 Mar 10;17(3):e0264838. doi: 10.1371/journal.pone.0264838 (PMC8912253; doi:10.1371/journal.pone.0264838)

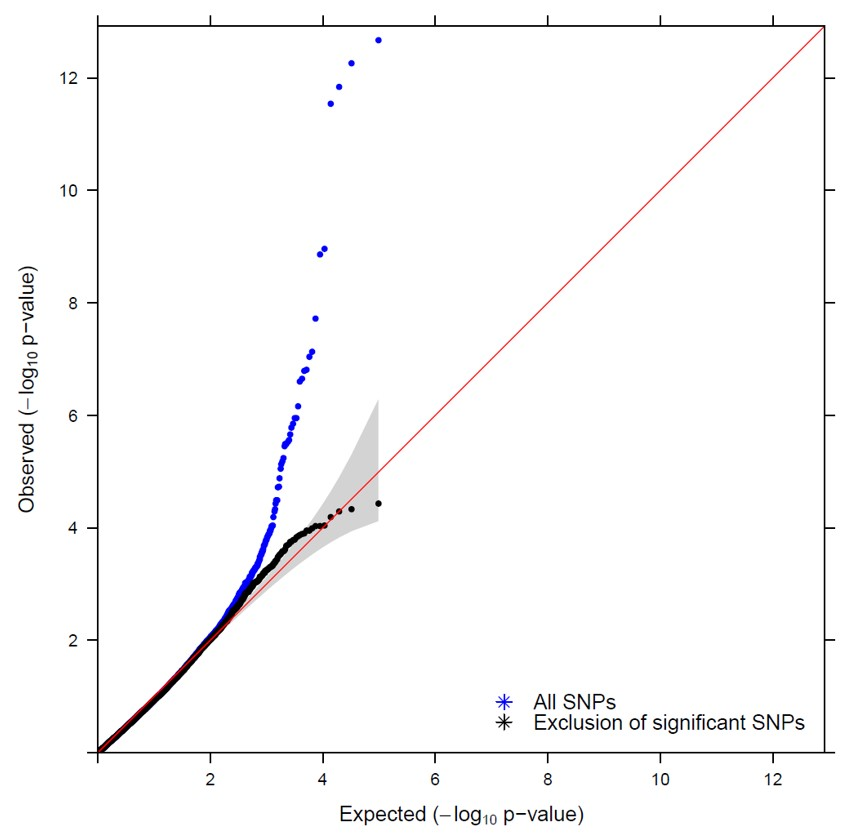

Supplement: S1 Fig — The sharp deviation above an expected -log10 p-value of approximately 3 is due to a strong association of 18-week body weight in turkeys with significant SNPs. Exclusion of significantly associated SNPs may leave a residual upward deviation leading to identify more associated SNPs with the trait, which was not the case in this study. (TIF) [file pone.0264838.s001.tif]

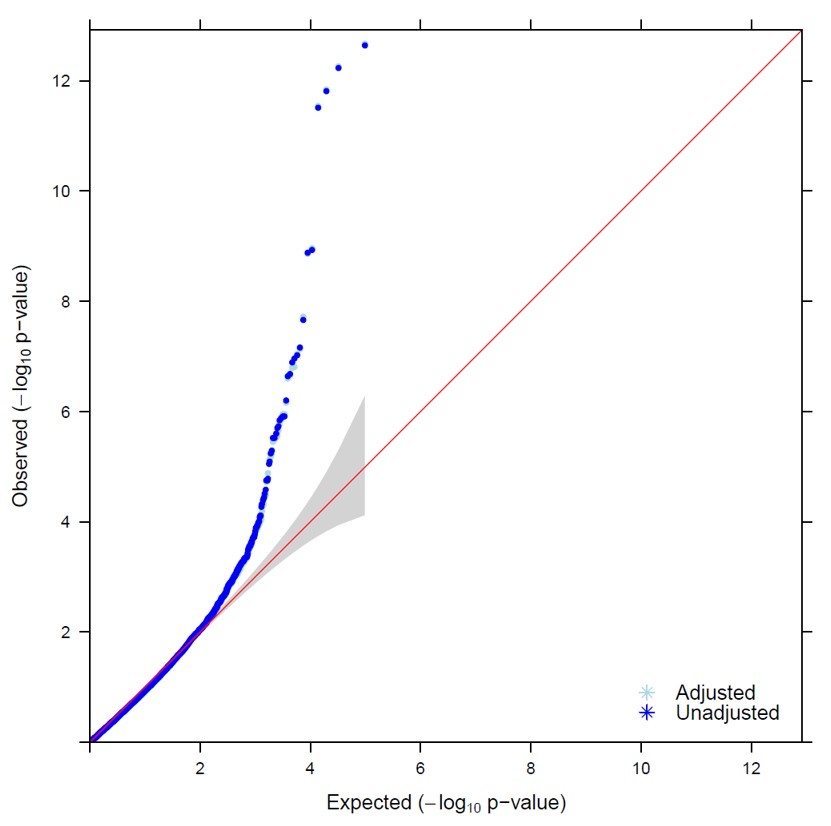

Supplement: S2 Fig — The adjustment for population stratification did not change the findings of this GWAS study. The population used in this study is a pure turkey line and confounding due to population subgroups is unlikely to be observed. (TIF) [file pone.0264838.s002.tif]
